# Improving Prediction of Postoperative Atrial Fibrillation After Cardiac Surgery Using Multiple Pathophysiological Biomarkers: A Prospective Double-Centre Study

**DOI:** 10.3390/jcm14113737

**Published:** 2025-05-27

**Authors:** Peter G. Noordzij, Maaike S. Y. Thio, Ted Reniers, Ineke Dijkstra, Gabriele Mondelli, Marloes Langelaan, Henk J. T. Ruven, Thijs C. D. Rettig

**Affiliations:** 1 Department of Anaesthesiology and Intensive Care, University Medical Centre Utrecht, 3584 CX Utrecht, The Netherlands; 2 Department of Anaesthesiology, Intensive Care and Pain Medicine, St. Antonius Hospital, Koekoekslaan 1, 3435 CM Nieuwegein, The Netherlands; 3 Department of Anaesthesiology, Intensive Care and Pain Medicine, Amphia Hospital, Molengracht 21, 4818 CK Breda, The Netherlands; 4 Department of Clinical Chemistry, St. Antonius Hospital, Koekoekslaan 1, 3435 CM Nieuwegein, The Netherlands; 5 Result Laboratory, Amphia Hospital, Molengracht 21, 4818 CK Breda, The Netherlands; 6 Department of Anaesthesiology and Intensive Care, Amphia Hospital, Molengracht 21, 4818 CK Breda, The Netherlands

**Keywords:** cardiac surgery, atrial fibrillation, biomarkers, risk stratification

## Abstract

**Background:** Postoperative atrial fibrillation (POAF) is a common and serious complication after cardiac surgery. Existing clinical prediction models show limited discriminative ability. We hypothesize that incorporating biomarkers that reflect key pathophysiological pathways of POAF can enhance preoperative risk stratification. **Methods:** Adult cardiac surgery patients without a history of atrial fibrillation from the BIGPROMISE cohort—a prospective, observational, two-centre perioperative biobank study—were included to investigate whether biomarkers of myocardial injury, systemic inflammation, haematological status, and metabolic and neuroendocrine dysregulation improved prediction of new-onset POAF when compared with an established clinical model, the POAF Score. We evaluated the incremental value of a 13-biomarker panel added to the POAF Score using multivariable logistic regression with shrinkage (lasso), assessing model discrimination, calibration, reclassification, and net clinical benefit. **Results:** Among 959 cardiac surgery patients, POAF occurred in 35% (n = 339). Inflammatory, metabolic, and neuro-endocrine biomarkers remained independently associated with POAF after applying lasso regression. Adding these biomarkers to the POAF Score improved discrimination, with the C-statistic increasing from 0.60 (95% CI: 0.60–0.60) to 0.63 (95% CI: 0.63–0.64; *p* < 0.01). Calibration was good in both models. At a threshold of 40% for high risk of POAF, the addition of biomarkers correctly reclassified 16% of patients with POAF as high risk. However, only 2% of the patients without POAF were reclassified as low risk, while 13% were incorrectly reclassified as high risk, resulting in a net reclassification index of 0.05. **Conclusions:** The addition of pathophysiological biomarkers significantly improves the performance of an established risk model for POAF after cardiac surgery, although the incremental clinical benefit is small.

## 1. Background

Postoperative atrial fibrillation (POAF) is the most common cardiac arrhythmia following cardiac surgery. Its incidence ranges from 15% to 50%, depending on the type of surgery and patient characteristics [1,2,3,4]. POAF is associated with increased postoperative morbidity, including stroke and heart failure [5,6]. While many episodes of POAF are transient and self-limiting, some patients develop persistent atrial fibrillation, which significantly worsens cardiovascular prognosis [7]. Preoperative risk prediction tools could aid in personalized prophylactic strategies aimed at reducing the incidence of POAF and associated complications, while limiting overtreatment.

A complex interplay of pathophysiological factors predisposes patients to atrial arrhythmias following cardiac surgery. Aging, chronic cardiac conditions, and metabolic and neuroendocrine disorders all contribute to POAF susceptibility, for example through atrial stretch and fibrosis, systemic inflammation, endothelial dysfunction, electrolyte imbalances, and oxidative stress [8,9]. The multifaceted nature of preoperative risk factors makes accurate prediction of POAF challenging. Despite extensive research, existing clinical risk models for POAF remain suboptimal, with moderate predictive performance at best [2].

The incorporation of predictors that represent the complex and heterogeneous nature of POAF may enhance individualized risk stratification and optimize preventive perioperative strategies, such as early initiation of beta-blockers, anti-inflammatory agents, or tailored electrolyte management. We hypothesize that a multimodal biomarker approach, using pathophysiological biomarkers for POAF, can significantly improve preoperative risk stratification, compared with clinical risk factors alone. This study aimed to investigate the added predictive value of a biomarker panel for POAF to the POAF Score, an established clinical prediction model for atrial fibrillation (AF) after cardiac surgery [1].

## 2. Methods

### 2.1. Study Design and Participants

This was a prospective, observational cohort study conducted at two cardiac surgery centres. Data were derived from the BIGPROMISE cohort, a perioperative biobank designed to investigate the epidemiology and pathophysiology of postoperative complications [10]. Eligible participants included adult patients who underwent cardiac surgery between 8 October 2021 and 9 September 2024. Individuals with a documented history of atrial fibrillation were excluded. The study was conducted in accordance with the Declaration of Helsinki and received approval from the institutional review boards of both participating centres [NCT05199025]. Written informed consent was obtained from all participants prior to enrolment. The study adhered to the TRIPOD (Transparent Reporting of a Multivariable Prediction Model for Individual Prognosis or Diagnosis) guidelines [11].

### 2.2. Data Collection

Demographic and clinical data were collected preoperatively for all participants in accordance with a prespecified study protocol [10]. Blood samples were collected after induction of anaesthesia, prior to the start of surgery. In all study patients, blood samples were centrifuged at 1800× *g* for 5 min and used to analyse a panel of 50 biomarkers [10]. Biomarker analyses were performed at the local hospital laboratory on Roche Cobas 8000 and Sysmex XN platforms. Results of perioperative biomarker analysis were captured in local laboratory information management systems and uploaded to a central web application for research data (REDCap (Nashville, TN, USA)).

### 2.3. Sample Size

Post hoc sample size calculation was performed to determine the maximum number of candidate predictors. Sample size estimation was informed by the pmsampsize package in R [12]. Sample size calculation was repeated with increasing numbers of candidate predictors using prevalence calculated from the cohort and expected discriminative model performance with a c-statistic of 0.72. The maximum number of candidate predictors for which the sample size was still sufficient was used for model development.

### 2.4. POAF Score

The primary objective of this study was to evaluate any increase in predictive value resulting from addition of a preoperative biomarker panel to the POAF score—an established and externally validated clinical prediction model for POAF in general cardiac surgery patients—compared with the POAF Score alone. We selected the POAF Score because it was derived and validated in a general cardiac surgery population and exhibits superior predictive performance compared with other available models. The POAF Score is a multivariable risk model incorporating age, history of chronic obstructive pulmonary disease, kidney function, urgency of surgery, preoperative use of an intra-aortic balloon pump, left ventricular ejection fraction, and surgery type [1].

### 2.5. Selection of Biomarkers

The initial biomarker selection was guided by existing literature on the pathophysiological mechanisms underlying POAF. These included myocardial injury, stress and fibrosis; inflammation; metabolic and neuro-endocrine dysregulation; and hematologic status [8,9]. To reduce the number of biomarkers from the initial selection to align with sample size constraints, univariable associations with POAF were assessed using the Mann–Whitney U test. This non-parametric, rank-based test was chosen to minimize the influence of outliers and accommodate the skewed distributions commonly observed in biomarker data. Biomarkers with the lowest *p*-values were included for model development.

### 2.6. Outcome

The primary outcome was new-onset POAF, defined as new onset of irregular heart rate in the absence of P waves lasting at least 30 s or for the duration of the ECG recording (if less than 30 s) [13]. Patients were monitored for the occurrence of POAF for up to 30 days postoperatively, or until hospital discharge, whichever occurred first. All participants underwent continuous cardiac telemetry for 48 h after CABG and 72 h after valve surgery, and standard 12-lead electrocardiograms (ECGs) were obtained on arrival at the intensive care unit, on postoperative days one and three, and on indication.

### 2.7. Missing Data

Missing data were assessed using descriptive statistics. Missing data were addressed using Multiple Imputation by Chained Equations (MICE), creating ten imputation sets with 20 iterations to minimize potential bias associated with complete case analysis. Variables with excessive missingness, defined as greater than 40%, were excluded from the analysis. Imputed datasets were pooled using Rubin’s Rules to derive final estimates.

### 2.8. Statistical Analysis

Baseline characteristics were summarized as mean ± standard deviation (SD) for normally distributed continuous variables or median with interquartile range (IQR) for non-normally distributed variables, as appropriate. Categorical variables were presented as counts with corresponding percentages. Differences between patients with and without POAF were assessed using Student’s *t*-test for normally distributed continuous variables, the Mann–Whitney U test for non-normally distributed variables, and the Chi-square test for categorical variables.

Least absolute shrinkage and selection operator (LASSO) regression was used for model development and variable selection. This method shrinks regression coefficients and excludes less informative predictors by setting some coefficients to zero, reducing overfitting of the model. For model development, the POAF Score was entered as a continuous probability and retained unpenalized to allow assessment of the incremental value of biomarkers. This analysis was repeated with the POAF Score penalized to evaluate whether biomarkers had a stronger association with the outcome than clinical variables. To meet model assumptions, multi-collinearity among candidate predictors was assessed. Correlation matrices were constructed, with a Pearson correlation coefficient (ρ) > 0.5 considered indicative of multi-collinearity. Independent variables were transformed as needed to meet the assumption of linearity with the log-odds of the outcome. LASSO was applied on each of the ten individual imputation sets, creating ten models. Predictors selected in at least five out of ten LASSO models were selected for the final model. LASSO regression was then performed again on all imputation sets using only the final predictor selection, creating the models used for model performance evaluation. For the Decision Curve Analysis (DCA) the mean of each coefficient of the imputation models was used to create a single final model; this was because, to the best of our knowledge, no statistical packages are available yet for DCA on multiple imputed datasets.

Model performance was evaluated through discrimination, using the area under the receiver operating characteristic curve (AUROC) with 95% confidence intervals (CIs); calibration, using calibration plots; reclassification, using the Net Reclassification Index (NRI) to assess the added value of biomarkers over the clinical model; and clinical benefit, using DCA to quantify the clinical utility of the prediction model according to a decision threshold. For NRI and DCA, a threshold probability of 40% was used. To quantify the difference in discriminative performance, a DeLong test was performed. Pooling was performed with the pfsmi package [14]. All statistical analyses were conducted using RStudio, version 4.4.0.

## 3. Results

### 3.1. Patient Population and Outcomes

The BIGPROMISE study cohort comprised 1180 adult patients undergoing cardiac surgery. Of these, 221 patients with a history of atrial fibrillation were excluded, resulting in 959 patients being included in the final analysis. Preoperative blood samples were missing in 14 patients (1.5%). Follow-up was complete for 100% of the cohort. The median age was 65.0 [59.0–70.0] years, and 757 patients (78.9%) were male.

In total, 339 (35.3%) patients developed POAF within 30 days following cardiac surgery. Patients who developed POAF were slightly older and underwent coronary artery bypass grafting (CABG) less frequently than those who did not develop POAF (Table 1). The incidence of POAF was 29.8% among patients undergoing isolated CABG and 36.3% among those undergoing single-valve surgery. Most patients developed POAF on postoperative days 2 (n = 129; 38.0%) and 3 (n = 94; 27.7%).

### 3.2. Biomarker Selection

An initial panel of 35 biomarkers was selected from the BIGPROMISE biobank. A complete overview of the candidate biomarkers and their respective pathophysiological domains is provided in Supplementary Table S1. Concentrations of the 13 biomarkers that were most strongly associated with POAF are presented in Table 2. Among the selected biomarkers, sex hormone-binding globulin (SHBG), along with NT-proBNP, cholesterol, and vitamin D, showed the strongest associations with the development of POAF.

### 3.3. Predictive Performance

**Figure 1:**
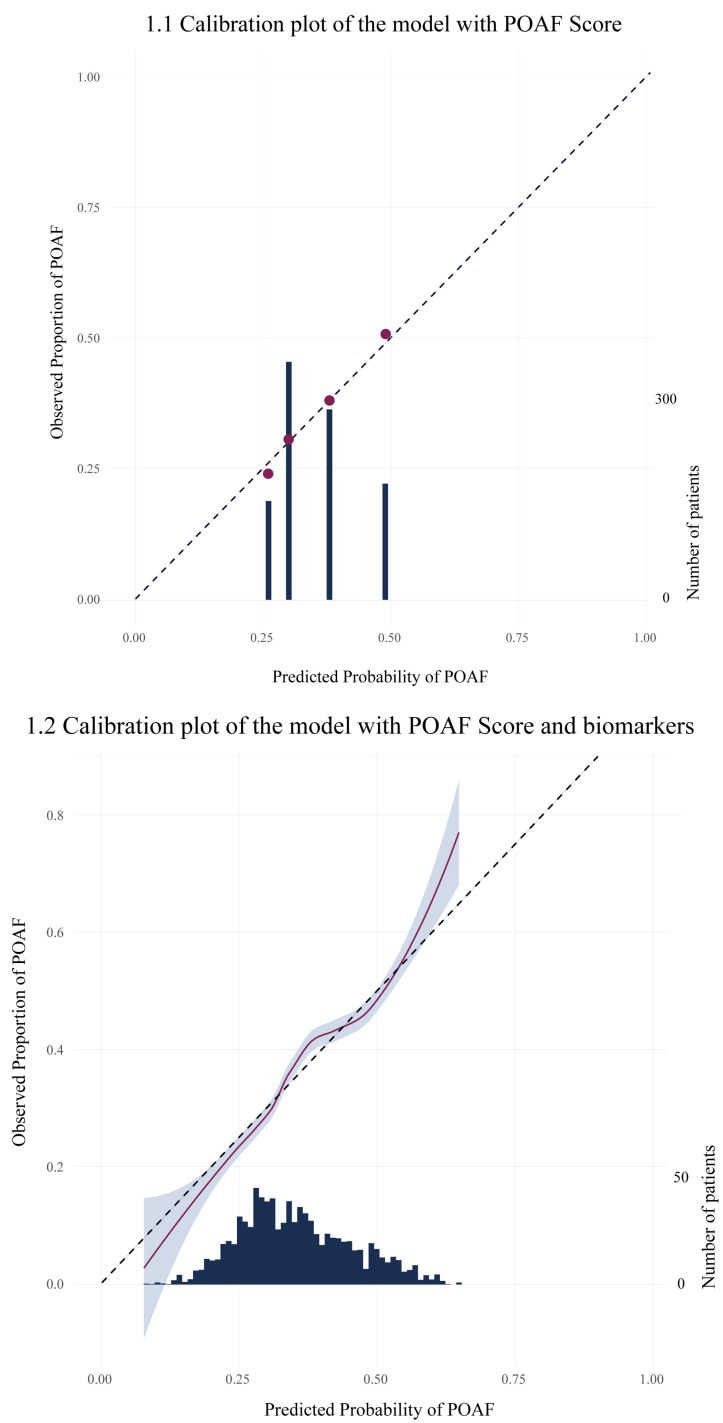
Calibration plots. (**1.1**) Calibration plot POAF Score; (**1.2**) Calibration plot biomarker model.

The discriminative ability of the clinical prediction model (POAF Score) was poor, with a C-statistic of 0.600 (95% CI: 0.596–0.604). The addition of biomarkers modestly, but significantly, improved model discrimination to a C-statistic of 0.633 (95% CI: 0.628–0.637; *p* < 0.01). Calibration was good in both models (Figure 1). However, both models slightly underestimated risk in low-risk patients and overestimated risk in high-risk patients.

### 3.4. Risk Stratification and Clinical Benefit

At a predicted risk threshold of 40%, the POAF Score identified 25.7% of patients with POAF as high risk. Incorporation of biomarkers into the model led to correct upward reclassification of 16% of patients who developed POAF. Two percent of patients without POAF were reclassified to a lower risk category, and thirteen percent were incorrectly reclassified as high risk. This resulted in a Net Reclassification Index (NRI) of 0.049 (CI −0.001–0.099) (Table 3 and Table 4), reflecting a very small improvement in risk classification.

**Figure 2:**
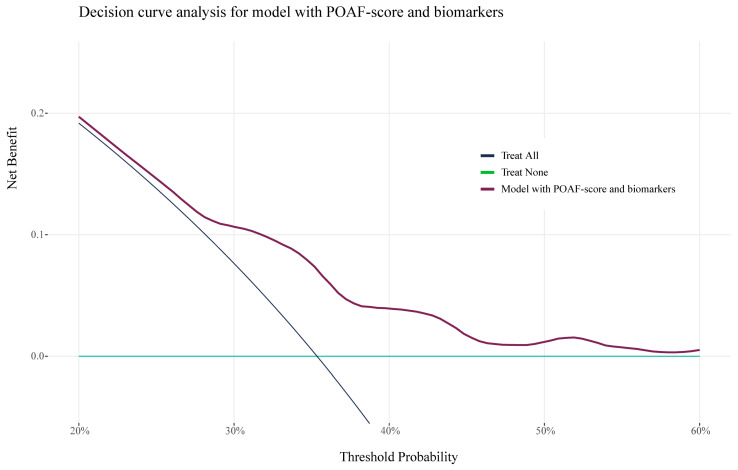
Decision curve analysis of biomarker-enhanced model.

Figure 2 presents the results of DCA, illustrating the net clinical benefit of the biomarker-enhanced model (blue line) for prophylactic treatment of POAF. Compared with a treat-all strategy, the biomarker enhanced model would avoid unnecessary treatment in 18% of patients, while maintaining treatment coverage for those who develop POAF. In comparison, the POAF Score alone would avoid unnecessary treatment in 17% of patients. When directly comparing the two models, the biomarker-enhanced model results in two fewer unnecessary treatments per 100 patients.

## 4. Discussion

This study aimed to improve preoperative prediction of POAF after cardiac surgery by adding a panel of pathophysiological biomarkers to an established clinical risk model. While the POAF Score is the best preoperative tool specifically developed for this purpose, its discriminative ability was poor in our cohort, a finding which was consistent with prior external validations [2]. We demonstrated that the addition of pathophysiological biomarkers modestly but significantly improved the predictive performance of POAF following cardiac surgery.

To strengthen the biological foundation of our model, all biomarkers were selected based on established or plausible pathophysiological pathways involved in POAF. Markers of myocardial injury, stress, and fibrosis, including GDF-15, hs-TnT, NT-proBNP, CK, and LDH, reflect cardiomyocyte damage and mechanical strain, which can lead to atrial remodelling and arrhythmogenesis. Inflammatory markers such as CRP, IL-6, leucocytes, ferritin, and—again—GDF-15 capture systemic inflammatory activity, a known trigger of atrial electrical instability after surgery. Neuroendocrine and metabolic biomarkers, including glucose, creatinine, electrolytes (potassium, sodium, calcium, magnesium), thyroid function tests (TSH, free T4), albumin, cholesterol, vitamin D, and sex hormone-binding globulin, were included for their influence on autonomic regulation, myocardial excitability, and systemic homeostasis. Finally, hematologic markers, such as haemoglobin, haematocrit, RDW, MPV, reticulocytes, thrombocytes, and leucocytes, reflect oxygen-carrying capacity, coagulation status, and inflammatory stress. This structured selection strategy ensured that the biomarker panel comprehensively represented the multifactorial and dynamic processes underlying POAF.

Although the overall improvement in discrimination of our biomarker-enhanced model was statistically significant, the incremental clinical value was limited. Given the poor predictive performance of the POAF Score alone (C-index 0.600), we anticipated that adding biomarkers would yield a more substantial improvement rather than a limited increase to 0.633. To further explore this, we repeated the analysis with the POAF Score penalized, allowing us to assess whether biomarkers had a stronger association with the outcome than clinical variables. Interestingly, even in this setting, the clinical predictors remained part of the final model, underscoring their robust predictive value despite the availability of a wide range of biomarker data. One might question the utility of a clinical prediction model with moderate performance. However, when used for risk stratification of prophylactic treatment for POAF our model could prevent unnecessary treatment in 18% of patients, compared with a treat-all strategy. Moreover, no clearly superior predictive models are currently available.

The limited addition in value resulting from the use of preoperative biomarkers in our model may be explained by the timing and context in which they were measured. Although biologically plausible, a single preoperative measurement may not adequately capture the dynamic, multifactorial processes that leads to POAF. Inflammatory and cardiac stress responses evolve rapidly during and after surgery, and preoperative biomarkers may not fully reflect the critical changes that precede the onset of POAF. Surgical trauma, extracorporeal circulation, fluid shifts, and temperature fluctuations likely contribute substantially to POAF by provoking inflammatory and autonomic responses [8,9]. Similarly, postoperative variables including blood loss, systemic inflammation, and haemodynamic instability are not captured in preoperative models, yet may carry significant predictive weight. This is supported by our finding that POAF development was most common on postoperative days 2 and 3. Incorporating postoperative biomarker measurements may therefore offer greater predictive value and enable earlier, more individualized clinical decision-making.

Future research is crucial to improve POAF prediction, given its impact on outcomes after cardiac surgery. Our findings suggest that preoperative biomarkers alone offer limited added value, likely due to the dynamic nature of POAF development. Future studies should explore the role of intra- and postoperative biomarkers to better reflect real-time pathophysiological changes. Advanced modelling approaches, including machine learning, may further enhance individualized risk stratification.

It is important to acknowledge several limitations. First, our biomarker-enhanced model was developed within a single study cohort without external validation. Second, biomarkers were collected after induction of anaesthesia, which may restrict generalisation to earlier preoperative settings. Third, dynamic intra- and postoperative factors were not included. Nevertheless, the strengths of this study include its prospective, multicentre design, systematic biomarker selection based on POAF pathophysiology and use of penalised regression techniques to reduce overfitting.

In conclusion, our findings demonstrate that adding pathophysiological biomarkers modestly improves preoperative risk stratification for POAF after cardiac surgery, with limited clinical benefit.

## Figures and Tables

**Table 1:** Baseline characteristics.

	No POAF	POAF	*p*-Value
	**N = 620**	**N = 339**	
Female, n (%)	126 (20.3)	76 (22.4)	0.498
Age, y (IQR)	64 [59–69]	66 [62–72]	<0.001
BMI	27 [25–30]	27 [25–29]	0.009
Surgery type, n (%)			<0.001
AVR	88 (14.2)	54 (15.9)	
Bentall	10 (1.6)	13 (3.8)	
CABG	393 (63.4)	167 (49.3)	
CABG + AVR	51 (8.2)	48 (14.2)	
MVR	42 (6.8)	20 (5.9)	
Other	36 (5.8)	34 (10.0)	
Urgent surgery	134 (21.6)	66 (19.5)	0.485
Diabetes, n (%)	154 (24.8)	70 (20.6)	0.541
COPD, n (%)			0.504
None	571 (92.1)	314 (92.6)	
GOLD I	3 (0.5)	4 (1.2)	
GOLD II	12 (1.9)	8 (2.4)	
GOLD III	6 (1.0)	4 (1.2)	
Unknown	28 (4.6)	9 (2.7)	
Hypertension, n (%)	344 (55.7)	187 (55.2)	0.935
Heart failure, n (%)	43 (7.0)	26 (7.7)	0.787
History of ischemic heart disease, n (%)	418 (67.6)	214 (63.3)	0.201
Previous myocardial infarction, n (%)	194 (31.3)	90 (26.5)	0.139
Myocardial infarction within 90 days prior to surgery, n (%)	128 (20.7)	58 (17.1)	0.207
Peripheral artery disease, n (%)	91 (14.7)	64 (18.9)	0.115
Pulmonary hypertension, n (%)			0.332
No	614 (99.0)	335 (99.1)	
Moderate	6 (1.0)	2 (0.6)	
Severe	0 (0.0)	1 (0.3)	
LVEF, n (%)			0.204
>50	460 (74.2)	260 (77.2)	
31–50	116 (18.7)	62 (18.4)	
21–30	14 (2.3)	8 (2.4)	
<20	7 (1.1)	0 (0.0)	
Unknown	23 (3.7)	7 (2.1)	
NYHA, n (%)			0.737
Class 1	132 (21.3)	82 (24.2)	
Class 2	270 (43.6)	150 (44.2)	
Class 3	66 (10.7)	29 (8.6)	
Class 4	11 (1.8)	6 (1.8)	
Unknown	140 (22.6)	72 (21.2)	
CCS IV, n (%)			0.256
No	509 (82.2)	287 (84.7)	
Yes	55 (8.9)	20 (5.9)	
Unknown	55 (8.9)	32 (9.4)	
Previous cardiac surgery, n (%)	75 (12.1)	34 (10.0)	0.386
Previous CVA or TIA, n (%)	70 (11.3)	39 (11.5)	1.000
Kidney function, n (%)			0.299
CC > 85	290 (46.8)	154 (45.4)	
CC 50–85	298 (48.1)	160 (47.2)	
CC < 50	30 (4.8)	25 (7.4)	
Dialysis	2 (0.3)	0 (0.0)	

AVR—aortic valve replacement; BMI—body mass index; CABG—coronary artery bypass grafting; CC—creatinine clearance; CCS—Canadian Cardiovascular Society; CVA—cerebrovascular event, LVEF—left ventricular ejection fraction; MVR—mitral valve replacement or repair; NYHA—New York Heart Association; TIA—transient ischemic attack.

**Table 2:** Baseline biomarker concentrations.

	No POAF	POAF	*p*-Value
	N = 620	N = 339	
SHBG (nmol/L)	32.00 [23.7–42.0]	35.8 [27.8–47.1]	<0.001
NT-proBNP (pg/mL)	176.8 [76.1–430.2]	228.3 [96.0–508.2]	0.012
Cholesterol (mmol/L)	3.6 [3.0–4.2]	3.7 [3.2–4.5]	0.016
Vitamin D (nmol/L)	45.0 [31.5–60.9]	49.0 [34.9–63.8]	0.024
Thrombocytes (×10^9^/L)	205.0 [173.0–237.0]	198.0 [169.0–226.0]	0.038
IGF-1 (nmol/L)	15.4 [12.0–19.1]	14.5 [11.7–18.0]	0.044
Glucose (mmol/L)	5.9 [5.5–6.8]	5.81 [5.40, 6.40]	0.044
IL-6 (pg/mL)	3.1 [1.9–4.1]	2.9 [1.9–3.6]	0.328
Red cell distribution width (%)	12.9 [12.4–13.4]	13.0 [12.5–13.5]	0.070
Reticulocytes (×10^9^/L)	61.0 [49.0–74.0]	58.3 [48.5–72.0]	0.088
Potassium (mmol/L)	3.9 [3.7–4.1]	3.9 [3.7–4.1]	0.181
Sodium (mmol/L)	139.5 [138.0–141.0]	139.9 [138.0–141.0]	0.200
GDF-15 (pg/mL)	1076.5 [779.5–1660.0]	1164.0 [858.5–1626.0]	0.186

NT-proBNP—N-terminal prohormone of brain natriuretic peptide; POAF—postoperative atrial fibrillation SHBG—sex hormone-binding globulin.

**Table 3:** Reclassification of cases at a risk threshold of 40%.

	Biomarker-Enhanced Model	
**POAF Score**	**<0.4**	**≥** **0.4**
**<0.4**	196	56
**≥0.4**	0	87

Values represent numbers of patients. Values in red represent cases that were incorrectly reclassified; values in green represent cases that were correctly reclassified.

**Table 4:** Reclassification of controls at a risk threshold of 40%.

	Biomarker-Enhanced Model	
**POAF Score**	**<0.4**	**≥** **0.4**
**<0.4**	454	82
**≥0.4**	9	75

Values represent numbers of patients. Values in red represent cases that were incorrectly reclassified; values in green represent cases that were correctly reclassified.

## Data Availability

The dataset supporting the findings of this study is available from the corresponding author upon reasonable request. Access to the data may be subject to institutional and ethical approvals, in accordance with applicable data-sharing policies.

## Supplementary Materials

The following supporting information can be downloaded at: https://www.mdpi.com/article/10.3390/jcm14113737/s1, Table S1: Biomarker selection according to the pathogenesis of postoperative atrial fibrillation.; Table S2: Model coefficients.
