# Supplementary material for: Improving Prediction of Postoperative Atrial Fibrillation After Cardiac Surgery Using Multiple Pathophysiological Biomarkers: A Prospective Double-Centre Study
Source: J Clin Med. 2025 May 27;14(11):3737. doi: 10.3390/jcm14113737 (PMC12156888; doi:10.3390/jcm14113737)
Supplement: Supplementary file 1 [file jcm-14-03737-s001.zip › jcm-3624284-supplementary.pdf]

Supplementary **Table S1.** Biomarker selection according to the pathogenesis of postoperative atrial fibrillation.

**Pathophysiologic pathway**	**Biomarkers**
Myocardial injury, stress and fibrosis	Growth differentiation factor-15, high-sensitive troponin T, N-terminal pro B-type natriuretic peptide, creatinin kinase, lactate dehydrogenase
Inflammation	C reactive protein, ferritin, growth differentiation factor-15, interleukin-6, procalcitonin, leucocytes
Neuroendocrine – metabolic	Albumin, calcium, cholesterol, ferritin, glucose, creatinin, magnesium, potassium, sex hormone binding globulin, sodium, thyroid stimulating hormone, free thyroxine, 25 hydroxyvitamin D
Haematological	Haemoglobin, haematocrit, red cell distribution width, mean platelet volume, leucocytes,<br>thrombocytes, reticulocytes

Supplementary **Table S2.** Model coefficients.

	**POAF-score unpenalized**	**POAF-score penalized**
Intercept	-2,48103195	-2,47648234
POAF-score probability	3,24666443	3,08268751
ln(SHBG)*	0,3318866	0,34396106
Cholesterol	0,09150807	0,09320337
Vitamin D	0,0021475	0,00224786
Thrombocytes^2*	-5,6871× 10^{-6}	-5,8366 × 10^{-6}
Glucose	-0,04823789	-0,04941777
IL6	-0,00740622	-0,00760614

*SHBG and thrombocytes concentrations were transformed for model development. An increase of 10 units in SHBG corresponds to approximately 2.30 points in the model (ln(10) ≈ 2.30). An increase of 10 units in thrombocytes corresponds to 100 points in the model (10^2.
